# Assessment of acute myocardial ischemia with unenhanced T1 mapping MR imaging

**DOI:** 10.1186/1532-429X-15-S1-P28

**Published:** 2013-01-30

**Authors:** Darach O h-Ici, Sarah Jeuthe, Thore Dietrich, Hubertus Pietsch, Gunnar Schuetz, Felix Berger, Titus Kuehne, Sebastian Kozerke, Daniel Messroghli

**Affiliations:** 1 Congenital Heart Disease and Pediatric Cardiology, Deutsches Herzzentrum Berlin, Berlin, Germany; 2 Internal Medicine-Cardiology, Deutsches Herzzentrum Berlin, Berlin, Germany; 3 Institute for Biomedical Engineering, University and ETH Zürich, Zürich, Switzerland; 4 Imaging Sciences and Biomedical Engineering, King's College London, London, UK; 5 MR and CT Contrast Media Research, Bayer Pharma AG, Berlin, Germany

## Background

Myocardial ischemia causes local edema. This has traditionally been studied using unenhanced T2-weighted MR imaging and contrast agent kinetic techniques. The aim was to study the acute T1 changes in-vivo in a novel closed chest animal model of myocardial ischemia.

## Methods

8 rats had an inflatable balloon coronary occluder surgically inserted via thoracotomy. They were allowed to recover for 10-14 days. MRI was performed to obtain baseline measurement of ventricular function. T1 mapping was performed using the Small-Animal Look-Locker Inversion Recovery (SALLI) technique.

Without removing the animals from the scanner, the left coronary artery was occluded for 30 minutes. Myocardial function and T1 were measured during ischemia.

MRI was performed on a whole-body 3.0-T MR unit with a 70 mm solenoid coil for rats. After generation of survey images and of a long-axis set of cine images, a stack of LV short-axis cine images was acquired to assess global function.

Short axis SALLI MR imaging was performed using the same short axis orientation in the mid ventricle, distal to the occluder. Typical SALLI parameters were as follows: 64 x 64 mm field of view, 0.60 x 0.60 mm pixel size, 3.0 mm-thick sections, 5.2/2.2, 10° flip angle, 4000 ms AD, 4000 ms RD, 12 phases (i.e., temporal resolution of 16.7 ms at 300 beats per minute), temporal undersampling factor of two, three signals acquired, and acquisition time of 8 minutes 30 seconds per slice. SALLI imaging was repeated throughout the duration of ischemia.

Images were transferred to a dedicated software package and T1 values were obtained for the left ventricle at baseline, and then for both the ischemic region of interest identified by myocardial hypokinesis and a remote zone unaffected by the coronary occlusion.

**Figure 1:**
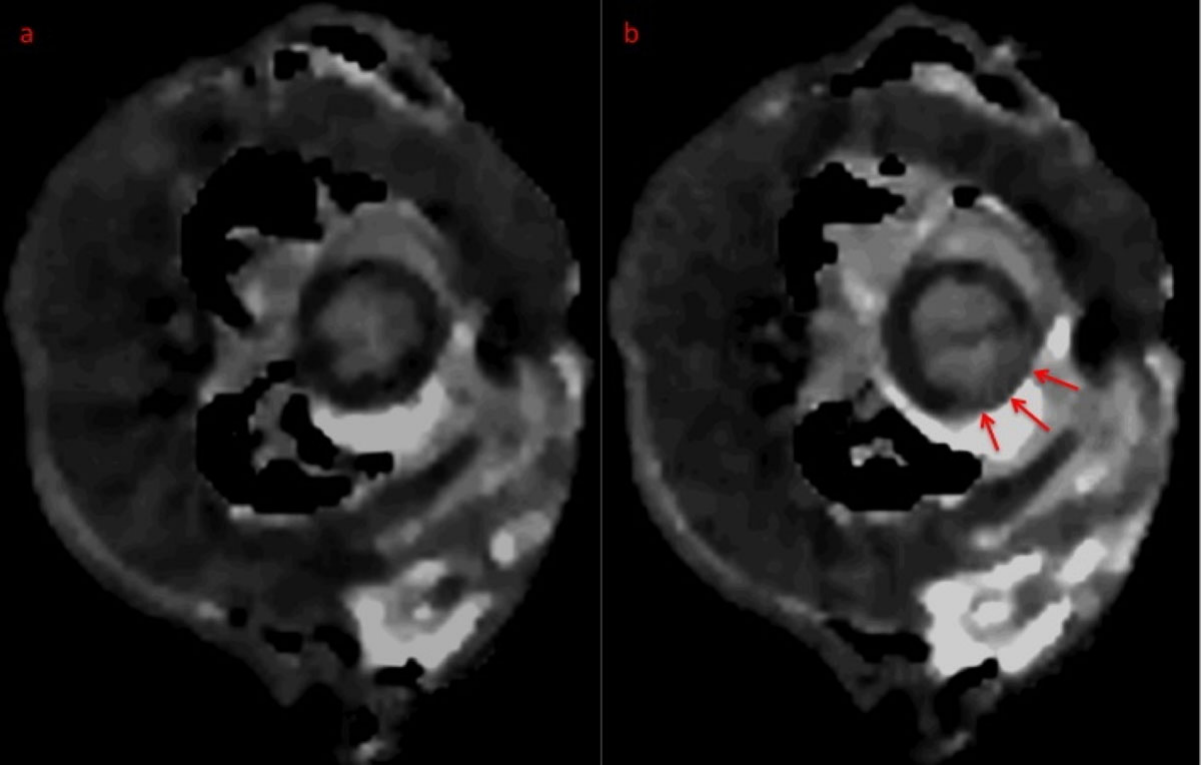
Representative midcavity short-axis T1 Maps at baseline (a, left) and during ischemia (b, right), demonstrating an area of prolonged T1 values (arrows) during ischemia.

## Results

All Rats had myocardial ischemia initially confirmed by ECG changes and ventricular arrhythmia. Fatal arrhythmia occurred in 2 rats.

T1 values increased from a baseline of 1102 +/- 76 to 1290 +/- 47 (p < 0.001) within the first 10 minutes of ischemia. T1 values remained unchanged during 30 minute period of ischemia (1290 +/- 47 vs 1294 +/- 70, p = 0.74).

## Conclusions

In rats, myocardial T1 changes occur within 10 minutes of ischemia following coronary occlusion. T1 values subsequently remain unchanged throughout a 30 minutes period of ischemia.

Unenhanced T1 mapping may provide a useful way to identify myocardial edema in acute myocardial ischemia/infarction, and may prove of value in studying the myocardial area at risk.

## Funding

Dr O h-Ici is funded through a "Sachmittelbeihilfe" granted to Dr Messroghli by the Deutsche Forschungsgemeinschaft. Drs Kuehne and Messroghli are supported through the German Federal Ministry of Education and Research (grants 01EV0704 and FKZ01G10210, 01GI0601).

